# Acceptability and performance of a directly assisted oral HIV self-testing intervention in adolescents in rural Mozambique

**DOI:** 10.1371/journal.pone.0195391

**Published:** 2018-04-05

**Authors:** Jonas Hector, Mary-Ann Davies, Johanna Dekker-Boersema, Mussa Manuel Aly, Cassimo Charifo A. Abdalad, Ernesto Belario Rafael Langa, Jochen Ehmer, Michael Andre Hobbins, Laura Frances Jefferys

**Affiliations:** 1 SolidarMed, Pemba, Mozambique; 2 School of Public Health and Family Medicine, University of Cape Town, Cape Town, South Africa; 3 Operational Research Unit Pemba, Pemba, Cabo Delgado, Mozambique; 4 District Health Directorate, Ancuabe, Cabo Delgado, Mozambique; 5 Provincial Health Directorate, Cabo Delgado, Pemba, Mozambique; 6 SolidarMed, Lucerne, Switzerland; The Ohio State University, UNITED STATES

## Abstract

**Introduction:**

Whereas progress in HIV testing and treatment has been made globally, the UNAIDS goal of “90 90 90” is still out of sight in rural northern Mozambique. New strategies that promote testing in hard to reach groups will aid Mozambique’s response to the HIV epidemic. HIV self-testing (HIVST) is recommended by the WHO as an additional approach to augment the HIV testing services available to adolescents. This study evaluates acceptability and performance of a directly assisted oral HIVST intervention for adolescents in rural Mozambique.

**Methods:**

Adolescents aged 16–20 years were included at schools and invited to attend the local hospital’s youth friendly service for directly assisted oral HIVST. Baseline and post-test questionnaires were obtained. OraQuick Rapid HIV-1/2 Anti body test^®^ was used. Results were read by the participant and by a nurse. Results were confirmed by finger prick HIV test (Determine^®^ HIV 1/2 Alere and Unigold^™^ HIV Trinity Biotech) according to the Mozambican national standard.

**Results:**

Between September and November 2016, 496 adolescents were included, of which 299 performed an oral HIV self-test. 70% were first time testers. The positivity rate was 1.7%. The inter-rater agreement between adolescent and nurse was 99.6% (kappa 0.93); there were no false negative or false positive results of the oral HIV self-test. Five tests were invalid. 7.1% found the test difficult to use. Over 80% preferred directly assisted HIVST compared to the standard finger prick testing. While 20% thought it would be good to do HIVST at home, 76% preferred to do HIVST at the health centre, for reasons including increased security, privacy, and the presence of a counsellor.

**Conclusions:**

Directly assisted oral HIVST is a feasible intervention for adolescents in rural Mozambique and showed encouraging results for first time HIV testers.

## Introduction

Sub-Saharan Africa (SSA) is disproportionally affected by the HIV epidemic [1]. Whilst progress in HIV testing and treatment has been made in the recent years, the UNAIDS goal of “90 90 90” is ambitious and may prove difficult to reach [2]. The WHO and UNAIDS call for an upscaling of current efforts in order to reach these goals [3]. In this context new service delivery strategies are necessary in order to target groups that are hard to reach by established services [4]. Adolescents (defined by the WHO as those aged 10–19 years) are a neglected group in HIV care and benefit less than other groups from established service provision models [5]. In SSA adolescents are less likely to be tested for HIV and linked to care. Furthermore in recent years the numbers of HIV related deaths in adolescents has risen, whereas in all other age groups HIV related deaths have fallen [5,6,7]. The WHO’s position paper on HIV and adolescent health calls for an improvement of service delivery [5]. A novel strategy to promote HIV testing is HIV self-testing (HIVST). The WHO recommends HIVST to be offered as a complementary approach to HIV testing services and emphasises the possible benefit of HIVST reaching hard to reach groups such as adolescents [8]. HIVST, as defined by the WHO, refers to the process of a person collecting their blood or oral fluid and performing and interpreting an HIV test themselves [8]. HIVST could either be directly assisted, which means the user receives assistance from a trained provider or peer before or during the process while unassisted HIVST is done alone only using instructions found in the test kit [8]. HIVST has been well studied in key populations in resource rich settings [9,10,11], and studies addressing HIVST in SSA settings have also been carried out [11,12,13,14]. However, there is a lack of understanding as to how HIV self-tests would perform in adolescents of rural populations. This study evaluates acceptability and performance of a directly assisted oral HIVST intervention in adolescents in rural Mozambique.

## Material and methods

### Setting

The study was set in two hospitals in the rural district of Ancuabe, Cabo Delgado Province, Mozambique. Both hospitals have a “one stop shop” youth friendly service (YFS), specifically designed for individuals aged 10 to 24 years. In the YFS adolescents and youth receive a complete package of health care services, thus avoiding the general outpatient clinic, general HIV testing services, and other services such as the laboratory, or pharmacy. The HIV prevalence in Cabo Delgado is rising and estimated to be 13.8% in the general adult population and 4.3% in the age group 15–20 years [15].

### Participants

Adolescents aged between 16 and 20 years were included in the study. Pregnant adolescents were excluded and referred to the antenatal clinic to be tested for HIV and complete their antenatal appointments.

Recruitment of the adolescents was done in the district’s two local secondary schools located in the catchment area of the two hospitals with YFS. Adolescents were also recruited directly in the two YFS of the hospitals in the district. Research assistants gave lectures on sexual and reproductive health (SRH) and HIV in the schools. The lectures were attended by all students above 14 years of age. Following the lectures, the study was introduced and students aged between 16–20 years were invited to participate in the study. Study participants each received a detailed individual explanation of the oral HIV self-test from the research assistant. The use of the oral HIV self-test was demonstrated by the research assistant, using a dummy oral HIVST kit. Participants were then given an invitation card to attend the YFS to perform the oral HIV self-test. Participants in the hospital were included by a research assistant according to the procedure at the schools, which included a lecture on SRH and HIV followed by study inclusion, detailed explanation of the oral HIV self-test, and a demonstration using a dummy oral HIVST kit.

### Study procedures

Oral HIVST was directly assisted; as instructions were given in person to each participant by the research assistant who explained how to use the oral HIV self-test [8]. The oral HIVST kits (OraQuick Rapid HIV-1/2 Anti body test^®^) with illustrated instructions in Portuguese were handed to the participants once they arrived at the YFS. Instructions on how to perform the oral HIV self-test were repeated (for adolescents who were included in the schools) by the research assistant if the participant wished. Participants performed the oral HIV self-test in the privacy of a separate room. After completion of the oral HIV self-test a nurse recorded the result as read and reported by the participant and as read by him/herself. The nurse then performed a confirmatory finger prick HIV test, (Determine^®^ HIV 1/2 Alere first-line assay and Unigold^™^ Trinity Biotech second-line assay [4]) according to the national standards and provided post-test counselling [16].

The oral HIV self-test was recorded as negative, positive, or, in the case of an absent or unreadable control line, invalid [17]. The finger prick HIV test was classified, in accordance with the national guidelines, as negative, positive, or indeterminate in the case of discordance between the Determine^®^ HIV 1/2 and Unigold^™^ HIV tests [16]. Each study participant completed a baseline questionnaire to capture demographic data, HIV risk perception, prior HIV testing, knowledge of health services, and the use of family planning. Study participants who attended the hospital for HIVST additionally completed a second post-test questionnaire to capture perceived acceptability of self-testing and whether there were any problems performing the oral HIV self-test. Questionnaires were filled out with the help of the two research assistants, who were unaware of the result of the HIV tests. Questionnaires were in Portuguese and both of the research assistants were also fluent in the local language in order to minimise any possible language barrier.

### Statistics

Data was entered into an Access database and analysed using Stata 13. Categorical data was compared using chi-squared test or Fishers Exact Test. Inter-rater agreement was evaluated by calculation of kappa. The continuous non-parametric data was compared using the Wilcoxon Rank Sum test.

Multivariate analysis was conducted to examine factors associated with attendance for directly assisted HIVST post invitation. Every variable for which there was an effect on HIV testing uptake reported in the literature was included in the final model. Results were expressed as Odds Ratios (OR) with 95% confidence intervals (CI). The qualitative data “determinants for choosing a testing site, risk perception, reasons for experienced difficulties, reasons for test preference” were categorized by the two authors JH and LJ to allow for quantitative analysis.

### Ethics

Each participant of the study gave written informed consent. Participants under the age of 18 provided assent to participate in the study. Parental permission was not obtained in order to maintain privacy and confidentiality of HIV testing and counselling. The study protocol, which addressed this important issue, was approved by the National Ethics Committee (Ref: 215/CNBS/16) in Maputo, Mozambique.

## Results

### Study participants

Between September and November 2016, 496 adolescents were included in the study.

372 students were included after SRH and HIV lectures given in schools and 124 adolescents were included directly at the youth friendly services at the hospital.

Baseline characteristics of the study participant are shown in Table 1.

**Table 1:** Baseline characteristic of the study participant (n = 496).

Girls (%)	193 (38.9)
Included at school (%)	372 (79.3)
Median age in years (IQR)	17 (16–19)
Knowledge about Youth friendly services (%)	20 (5.4)^a^
Using any method of family planning (%)	200 (40.3)
Condom (%)	112 (22.6)
Oral contraceptive (%)	19 (9.8)^b^
Injectable hormonal (%)	69 (35.8)^b^
Currently attending school (%)	433 (87.3)
Previously tested for HIV (%)	148 (29.8)
Perception of being at risk of HIV (%)	211 (42.6)

^a^ Calculated with n = 372, corresponding to the number of adolescents that were included in the schools,

^b^ Calculated with n = 193, to the number of female study participants.

### Access to sexual and reproductive health care services

The vast majority (95%) of the adolescents, who were included at school, were not aware that a youth friendly service existed at their local hospital. The use of family planning was reported by 40% of the participants, however only 22.6% reported using condoms. About one third (29.8%) of participants reported previously testing for HIV, this being was more common in girls than boys (46.7% vs 19.1%; p<0.001). Less than half (42.6%) of the participants perceived themselves to be at risk of contracting HIV. This perception was more common in boys than girls (46% vs 28.6%, p = 0.002). Among adolescents who felt they were not at risk of HIV just 39.3% could correctly justify this perception by either stating that they used condoms (23.6%) or were abstinent (15.7%). The remaining 60.7% of adolescents that stated they were not at risk of HIV demonstrated a limited understanding of HIV risk and gave reasons such as having only one sexual partner (8.5%), trusting their sexual partners (6.9%) or being confident about their own good health (45.3%).

### Oral HIV self-test

Of the 372 adolescents included in the schools, 175 (47%) came to the hospital, received pre-test counselling, performed an oral HIV self-test, and received an HIV validation test and post-test-counselling. All of the 124 adolescents included in the hospital received pre-test counselling, performed an oral HIV self-test, received an HIV validation test and post-test-counselling. In total, 299 adolescents performed oral HIV self-tests.

Table 2 depicts the odds ratio of attending the hospital for directly assisted oral HIVST after receiving an invitation at school. In the univariate analysis adolescents who were using family planning or who had knowledge about the YFS were more likely to attend the hospital for directly assisted oral HIVST. However in the multivariate analysis these factors lost significance.

**Table 2:** Univariate and multivariate analysis of factors associated with attendance for directly assisted oral HIVST post invitation (n = 372).

Characteristics of the adolescents	Odds ratio of conducting the HIV self-test after invitation (95% CI)	
	Univariate analysis	Multivariate analysis
Female	1.45 (0.93–2.27)	1.30 (0.79–2.13)
Age ^a^	0.94 (0.81–1.09)	0.91 (0.77–1.06)
Prior HIV test done	1.15 (0.72–1.81)	0.93 (0.55–1.59)
Perception to be at risk	1.14 (0.75–1.73)	1.37 (0.88–2.13)
Use of family Planning	1.61 (1.05–2.48)	1.54 (0.95–2.5)
Knowledge about Youth friendly services	2.76 (1.03–7.42)	2.46 (0.89–6.92)

^a^ Odds ratio expressed for each year of increasing age.

Table 3 shows the results of the oral HIV self-test as read by the adolescent and read by the nurse as well as the result of the confirmatory finger prick HIV test. For the oral HIV self-test, the interrater agreement between the adolescent and the clinician was 99.6%. (kappa 0.93). There were no false positive or false negative results of the oral HIV self-test and the HIV positivity rate was 1.7%. However for six participants there was either discordance in the interpretation of the HIV self-test result or in the confirmatory test result.

**Table 3:** Result of the oral HIV self-test and the confirmatory HIV test.

Result	HIV self-test read by adolescent	HIV self-test read by clinician	Standard HIV-test
Positive	5	5	5
Negative	290	289	291
Invalid ^a^	4	5	NA
Indeterminate ^b^	NA	NA	3

NA = Not applicable.

^a^ The oral HIV self-test was invalid if no control line was visible.

^b^ The standard HIV test was indeterminate if results were discordant between first line assay (Determine^®^ HIV1/2) and second line assay (Unigold^™^ HIV).

One oral HIV self-test, which was read as negative by the adolescent, was read as invalid by the clinician and resulted in an indeterminate confirmatory HIV test. A second negative oral HIV self-test, read correctly by the adolescent, resulted in an indeterminate confirmatory HIV test. Four oral HIV self-tests, were read as invalid by the adolescents and the nurse and resulted in three negative confirmatory HIV tests and one indeterminate confirmatory HIV test.

Of the 175 adolescents included in the schools who had received a demonstration of the oral HIV self-test with a dummy kit, 25 (14.3%) said they would be able to perform the test at the clinic without a repeated explanation. 253 adolescents (85%) preferred directly assisted oral HIVST to the standard HIV testing, but a few adolescents (7.1%) found it difficult to perform the oral HIV self-test. The principal reason for preferring directly assisted oral HIVST was that no finger prick was needed to perform the test (56.4%). The oral HIV self-test was easy to use for 38% of the adolescents and 5.6% said they found directly assisted oral HIVST to protect their privacy.

**Fig 1:**
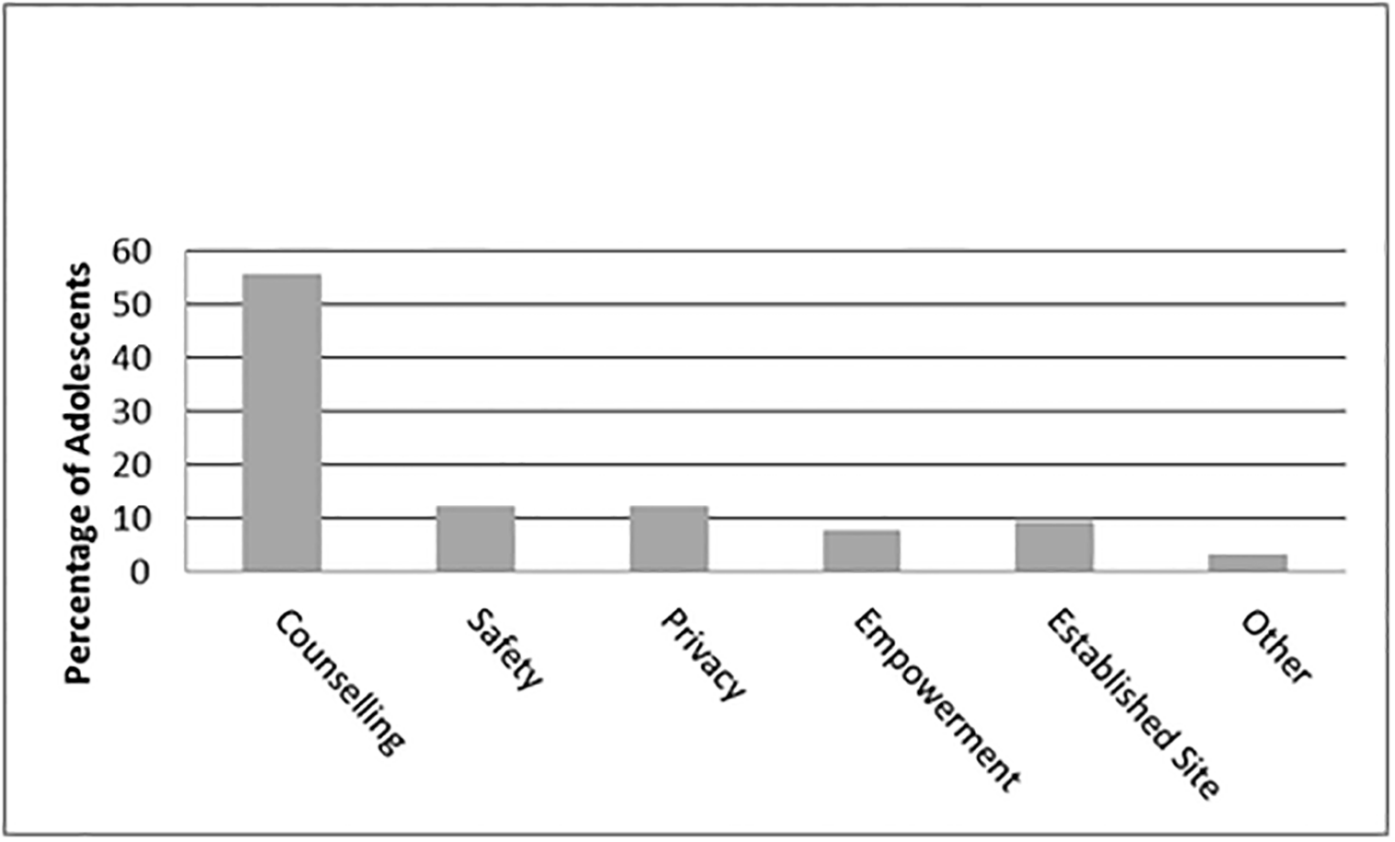
Determinants for choosing a testing site.

The primary reason offered by 40 (15%) adolescents who preferred the standard HIV test was that they trusted the result over that of the HIV self-test, stating that “HIV is in the blood, therefore blood needs to be tested to detect HIV”. 234 (78.2%) study participants thought it would be possible for them to do the oral HIV self-test unsupervised at home. However, when asked for the most appropriate place for HIVST, only 67 (22.4%) stated they would prefer to do oral HIVST at home and only four (1.3%) stated they would prefer to do oral HIVST at school. The majority (228, 76.3%) responded that they would prefer to do oral HIVST at the hospital in the YFS. Reasons why adolescents chose specific locations for oral HIVST are illustrated in Fig 1.

## Discussion

Adolescents in this study found the oral HIV self-test easy to use, this is in line with other studies where the majority of adolescents only required one or two explanations prior to performing the test [18,19]. Some adolescents participating in this study were concerned regarding the accuracy of the result obtained from oral HIVST, as shown in other studies from similar rural settings in India and Tanzania [19,20]. However, in accordance with other studies from SSA, we found the majority of participants preferred oral HIVST compared to the blood based test, due to the absence of a finger prick [18,19,21]. This study attracted first time testers, with 71% of participants testing for HIV for the first time. A literature review by Krause et al, which reviewed 11 HIVST studies, noted that HIVST encouraged first time testers [22]. This supports the WHO’s position on HIVST as a complementary method to reach adolescents who aren’t accessing established HIV testing services. In addition to receiving HIV testing, adolescents can also benefit from accessing health care services, receiving condoms, family planning, and regular HIV testing [16]. This is especially important in countries such as Mozambique where a cultural acceptability of premature marriage and pregnancy can put adolescents at risk of contracting HIV or suffering from the socio-physiological impact associated with teenage pregnancy [23,24].

Five adolescents in the study were HIV positive, corresponding to an observed positivity rate of 1.7%. This is lower than the 4.3% HIV prevalence in the age group 15–19 years in the province of Cabo Delgado [15]. The positivity rate in our study is likely to be underestimated as it was not designed to include a representative sample of the adolescent population of the district, and it is unlikely that adolescents who knew they were HIV positive would volunteer to participate in the study. There were no false positive or negative oral HIV self-test results. However, 5 invalid oral HIV self-tests were observed in our study (1.7%). Invalid test results are a common problem in HIVST and rates vary between setting and implementation of HIVST. A study by Ng et al in Singapore found three invalid HIV self-tests from 986 (0.3%) tests which is a much lower rate than observed in our study [25]. Kurth et al reported a higher rate of invalid HIV self-tests (15.5%) in their study of 239 participants in Kenya. [26]. Reasons for invalid HIV self-tests could be manifold and our study can’t determine the causes of the five reported invalid tests. The literature describes issues of sample collection, as well as the handling of the sample as possible sources of error [26, 27]. In light of the 1.7% invalid tests we think that directly assisted oral HIVST is the best option for oral HIVST in rural Mozambique, as the presence of a counsellor can mitigate any fearful reactions triggered by an invalid (or positive) test result. A counsellor can also provide HIV and sexual health information to people attending testing services, a further reason to recommend directly assisted HIVST, as the WHO recommends HIV testing services to be accompanied by sexuality education [5].

Schools can play an important role in linking adolescents to health care services [28]. However it has also been shown that the success of HIV testing outside of health facilities is very context specific; with acceptability varying between settings [29,30,31]. In our study the school was not a preferred location for testing, however using the schools as a platform to motivate adolescents to test in the health facilities was found to be acceptable and effective. Additionally, the home was not considered a desirable location for oral HIVST, this is in contrast to other studies from SSA [32,33,34]. In a rural setting where daily activities are conducted in the open and homes are simple structures of mud, bamboo, and grass [35] used primarily for sleeping, it is understandable why many participants may have viewed the health centre as a more private and safer place to perform an oral HIV self-test.

Although privacy and safety were important, the main reason given by the adolescents to test in the health centre, was the presence of a trained counsellor. This indicates that for similarly developed rural settings directly assisted HIVST in a health facility is probably more appropriate than unassisted HIVST outside of the health facility.

A limited knowledge of HIV transmission was demonstrated by some of the adolescents in our study when exploring perceived HIV risk. This observation is in line with other studies, which demonstrated a great heterogeneity in perceptions of HIV risk where some adolescents perceived their risk of contracting HIV as low even if engaging in risky behaviour [36,37]. This demonstrates the vulnerability of adolescents to contracting HIV and other sexually transmitted infections.

We also found that the rate of condom use of the adolescents that participated in our study was low. Recent data from a cross-sectional survey showed only 41% of women and 67% of men of reproductive age in Cabo Delgado knew that condom use can prevent HIV and that only 10% of men who had sex with more than one woman in the past year had used a condom during their last sexual encounter [38]. Another study in Mozambique noted that condom use was much lower in the northern rural regions of the country with and estimation of only 6% of men of reproductive age using condoms [39]. Besides complex behavioural reasons for low condom use,[40,41] a study by Wagenaar et al evaluated availability of condoms in Mozambique and found that health centre stock outs occurred frequently with more stock outs occurring as the distance between health centre and district warehouse increased [42].

### Limitations

The majority of our study population were students and thus do not represent the general adolescent population. The HIV positivity rate from this study should therefore be examined critically. Due to the expected low number of positive HIV tests this study was not powered to calculated sensitivity and specificity of the oral HIV self-test. Students have better access to sexual reproductive health education in comparison to out of school youth. Therefore, knowledge about HIV and uptake of testing and counselling could be considerably lower in out of school youth. Participants chose to attend the health centre and perform the oral HIV self-test, thus introducing a selection bias and acceptability of directly assisted oral HIVST could therefore be overestimated. Questionnaires were designed with local study assistants and in conjunction with local adolescents however, cultural norms could have prevented participants from openly expressing their opinions. We did not detect any statistically significant factors that predict attendance at the hospital for directly assisted oral HIVST after giving an invitation. We do not exclude the possibility that a larger sample size could have detected statistically significant factors.

## Conclusions

Sensitization in schools in conjunction with the novel strategy of directly assisted oral HIVST encouraged adolescents, especially first time testers, to be tested for HIV. Participants preferred to perform the oral HIV self-test in the health centre and when given the choice preferred directly assisted oral HIVST to the standard finger prick test. Directly assisted oral HIVST at the health centre has been shown to be both feasible and acceptable for adolescents in this extremely rural setting. In general, the study participants were not in favour of unassisted HIVST at home or at school, a fact that should be considered if rolling out HIVST services in similar rural settings.

## Supporting information

S1 Dataset (XLS)
